# Manipulation of miRNA activity accelerates osteogenic differentiation of hMSCs in engineered 3D scaffolds

**DOI:** 10.1002/term.435

**Published:** 2011-06-27

**Authors:** Peter D Mariner, Erika Johannesen, Kristi S Anseth

**Affiliations:** 1 Department of Chemical and Biological Engineering, University of Colorado at BoulderCO, USA; 2 Howard Hughes Medical Institute, University of Colorado at BoulderCO, USA

**Keywords:** microRNA (miRNA), human mesenchymal stem cell (hMSC), osteogenesis, bone, poly(ethylene glycol) (PEG)

## Abstract

Cell-based tissue engineering strategies have shown tremendous promise for the repair of bone mass deficiencies, but the efficient and appropriate induction of stem cells down osteogenic pathways remains a significant roadblock to the effective implementation of cell-based therapies. When grown in culture, human Mesenchymal Stromal/Stem Cells (hMSCs) remain multipotent, requiring specific exogenous signals to induce osteogenic differentiation. hMSCs used in transplantations, therefore, must be presented with local signals, often provided by the host's own tissues, to be directed down bone-related lineages. This process is relatively inefficient and remains difficult to control. In an effort to enhance osteogenesis, hMSCs were transfected with specific miRNA mimics and inhibitors that had originally identified for their ability to increase Alkaline Phosphatase (ALP) activity. Transfection with miRNA reagents had the effect of sensitizing hMSCs to soluble osteogenic factors, resulting in a rapid and robust induction of bone-related markers, including ALP activity and calcium deposition. Synthetic 3D tissue constructs prepared with miRNA-transfected hMSCs demonstrated similar responses to soluble osteogenic signals, suggesting that controlling miRNA activity in hMSCs can be an effective tool for enhancing the induction of osteogenesis for tissue engineering purposes. Copyright © 2011 John Wiley & Sons, Ltd.

## 1. Introduction

Current clinical strategies for repairing bone mass defects have inherent limitations that have driven the development of new tissue engineering approaches to employ the osteogenic potential of bone-derived mesenchymal stromal/stem cells (MSCs). Originally isolated and described by Friedenstein *et al.* (1966), MSCs can be cultured *in vitro*, allowing for the rapid expansion of multipotent cells (Schallmoser *et al.*, 2008) that are capable of differentiating into several distinct cell types, including osteoblasts, chondrocytes, adipocytes and myocytes (Pittenger *et al.*, 1999). In cell culture, MSCs derived from adult human bone marrow (hMSCs) can be induced down osteogenic pathways by the inclusion of dexamethasone, β-glycerol phosphate and ascorbate-2-phosphate in the culture media (Jaiswal *et al.*, 1997>). Given this potential, numerous experiments were initiated to test the efficacy of MSCs to induce *in vivo* bone regeneration in animals, and pioneering studies in rats (Ohgushi *et al.*, 1989), mice (Krebsbach *et al.*, 1998), dogs (Bruder *et al.*, 1998) and sheep (Kon *et al.*, 2000; Petite *et al.*, 2000) showed significant promise and led to countless follow-up studies. The success of these animal studies opened the door to multiple clinical trials in which stem cells have been used to treat bone mass deficiencies (reviewed in Arthur *et al.*, 2009; Bajada *et al.*, 2008). Reconstructive surgeries that employed the use of these autologous stem cells in mandibular and cleft palate defects, for example, showed enhanced bone formation and improved union healing (Hibi *et al.*, 2006a, 2006b; Kitoh *et al.*, 2004; Velardi *et al.*, 2006; Warnke *et al.*, 2004).

Although MSC-based tissue engineering strategies have shown tremendous promise for the regeneration of bone mass defects, the efficient induction of osteogenesis in these cells remains a significant roadblock to the effective implementation of cell-based therapies. When grown in culture, MSCs remain multipotent, requiring specific exogenous signals to induce osteogenic differentiation. For MSCs used in transplantations, osteoinduction is generally accomplished by local signals provided by the host's own tissues, though this remains difficult to control and relatively inefficient. For this reason, osteoinductive signals have been included in material scaffolds that are used to deliver MSCs to defect sites, providing additional stimuli for directing tissue regeneration (Marion and Mao, 2006). Alternatively, others have genetically engineered MSCs to overexpress osteoinductive signals, such as bone morphogenetic protein-2 (BMP-2) (Lindsey, 2001), growth factor or core-binding factor-α 1A (CBFA1) transcription factor (also known as Runx2) (Zhao *et al.*, 2007). While each of these techniques has shown promise, the recent discovery of microRNAs (miRNAs) and their ability to control global gene expression patterns has offered a potential strategy for directing stem cell differentiation down specific developmental lineages and dramatically improving MSC-based techniques for bone regeneration.

miRNAs are short, non-coding RNA molecules that act to inhibit the expression of target mRNAs and serve important regulatory roles in developmental processes (reviewed in Erson and Petty, 2008; Lee *et al.*, 2006). When expressed, these small RNAs are processed and incorporated into larger riboprotein complexes that can target multiple messenger RNAs (mRNAs), shortening the half-lives of the mRNAs and/or preventing their translation into functional proteins. Targeting of these riboprotein complexes to specific mRNAs is accomplished by sequence-dependent interactions between the miRNA and the 3′ untranslated regions of the mRNAs, allowing miRNAs to inhibit the expression of multiple genes containing the same binding motif. Often, the genes that are targeted by each miRNA function in similar metabolic pathways, allowing miRNAs to coordinately regulate global gene expression patterns and control cell fates.

As the field of miRNAs emerged, it became evident that miRNA expression profiles are cell type-specific and that miRNAs likely play a significant role in controlling cellular differentiation (Lee *et al.*, 2006). Because miRNAs can target a wide range of seemingly unrelated mRNAs, they possess a unique ability to regulate global gene expression patterns that ultimately determine cell function. Moreover, recent comparisons between stem cells and cells that are terminally differentiated indicate that miRNA expression profiles become more and more complex as cells commit to different cell lineages (Nie *et al.*, 2008; Strauss *et al.*, 2006), leading us to hypothesize that the controlled manipulation of miRNA activity in hMSCs could promote osteogenesis and enhance tissue-engineering strategies that involve the use of stem cells for regenerating bone.

In a recent study to demonstrate the efficacy of their miRNA products, Schoolmeesters *et al.* (2009) from Thermo Fisher Scientific screened a library of miRNA mimics and inhibitors to identify miRNAs reagents that induce alkaline phosphatase (ALP) activity. In this study, hMSCs transfected with either a mimic of miRNA-148b (M-miR148b) or an inhibitor of miRNA-489 (I-miR489) increased ALP activity after 6 days when compared with cells that received non-targeting miRNA controls. Because ALP activity is considered a hallmark of osteogenesis, these results suggested that the manipulation of miRNA activity in stem cells could be used to direct differentiation and raised the question whether this approach could be used for tissue-engineering purposes. *In silico* analysis identified a multitude of potential gene targets for these miRs, including a subset of genes related to MSC differentiation and BMP signalling (Schoolmeesters *et al.*, 2009). Although additional work will need to be done to clearly define the direct targets of miR-148b and miR-489, these miRNAs have been shown to be differentially regulated in osteoblasts exposed to specific graft materials (Palmieri *et al.*, 2007, 2008), and their ability to affect osteogenesis in MSCs appears to be clear.

In the work presented here, we demonstrate that the transfection of hMSCs with a miR-148b mimic and a miR-489 inhibitor acts to sensitize these cells to exogenous osteogenic signals and greatly enhances the induction of bone-related tissue markers in both two-dimensional (2D) and engineered three-dimensional (3D) environments. This study demonstrates that controlling miRNA activity in hMSCs can be an effective tool for enhancing the induction of osteogenesis and provides insight into the tremendous potential that targeting miRNA activity can have on the field of tissue engineering.

## 2. Materials and methods

### 2.1. hMSC culture

Human mesenchymal stem/stromal cells (hMSCs) were isolated from bone marrow aspirates (Cambrex) by their adhesion to standard tissue culture polystyrene plates incubated at 37 °C and 5% CO_2_. Briefly, aspirates were aliquoted onto 10 mm plates containing 10 ml control medium (CON) [low-glucose Dulbecco's modified Eagle's medium (DMEM; Invitrogen), supplemented with 10% fetal bovine serum (FBS; Invitrogen), 10 U/ml penicillin (Invitrogen), 10 µg/ml streptomycin (Invitrogen) and 0.5 µg/ml fungizone]. After 72 h, each plate was washed with CON medium to remove non-adherent cells. The remaining adherent cells were cultured in CON medium until significant cell numbers were observed, at which point the cells were trypsinized off the plates and frozen until used in experiments. Isolated cells showed multilineage potential by their ability to differentiate down osteogenic, chondrogenic and adipogenic pathways (data not shown). For each experiment, hMSCs were thawed from frozen stocks and expanded in CON medium supplemented with 1 ng/ml fibroblast growth factor (R&D Systems, Minneapolis, MN, USA). Cells used in transfections were taken from either passage 2 or 3, with no discernible difference in their response to treatments observed between passages. After transfection, cells were maintained in CON medium overnight before being treated with specified media conditions. Osteogenic medium (OST) was prepared by supplementing CON medium with 100 nm dexamethasone (Sigma, St. Louis, MO, USA), 50 µm ascorbic acid-2-phosphate (Sigma) and 1 mm β-glycerol phosphate (Sigma) (Jaiswal N, *et al.*, 1997). For 2D studies, hMSCs were seeded into 96-well plates at a density of 2 × 10^4^ cells/cm^2^. For 3D studies, hMSCs were photoencapsulated (5 × 10^6^ cells/ml) in hydrogels consisting of 6% w/v 20 K four-arm poly(ethylene glycol)–norbornene (4-arm PEG-NB), 1.0 mm adhesion peptide (CRGDS) and MMP-degradable peptide crosslinkers (KKCGGPQGIAGQGCKK) in 1× PBS (Fairbanks *et al.*, 2009a, 2009b). Briefly, hMSCs were resuspended in the monomer solution containing 0.01% of the photoinitiator lithium phenyl-2,4,6-trimethylbenzoyl phosphinate (LAP). 30 µl of the cell suspension was then placed in the top of a cut-off 1 ml syringe and exposed to 4 mW/cm^2^ of 365 nm light to initiate polymerization. After 4 min, the cell-encapsulated hydrogels were transferred to tissue culture plates containing CON medium. After overnight incubation in CON medium, the hydrogel constructs were transferred to 24-well plates containing treated media conditions.

### 2.2. hMSC transfection with miRNA mimics and inhibitors

hMSCs were transfected with commercially-available miRNA mimics and inhibitors (miRIDIAN, Dharmacon/ThermoFisher, Lafayette, CO, USA) using the Human MSC Nucleofection Kit from Lonza (Walkersville, MD, USA) (Tuli *et al.*, 2003). Briefly, trypsinized and pelleted hMSCs were resuspended in MSC nucleofection buffer at a concentration of 2.5 × 10^6^ cells/100 µl transfection. miRNA mimics and inhibitors (0.1 nm each) were then added to the cells and incubated at room temperature for 2–3 min. Cell–miRNA suspensions were placed into cuvettes and transfected with the U-23 programme. Cells were transferred into RPMI medium containing 10% FBS (Invitrogen) and allowed to recover for 20–30 min at 37 °C and 5% CO_2_ before being either plated or encapsulated. Expression levels for each miR was measured by qRT–PCR, using TaqMan-based primer sets purchased from Ambion (Austin, TX, USA) according to the manufacturer's instructions.

### 2.3. Alkaline phosphatase (ALP) assays

For 2D studies, cells seeded in 96-well plates were washed once with 1× PBS before being lysed with 50 µl of 1× RIPA buffer (Upstate/Millipore, Temecula, CA, USA). Cell lysates were diluted with 150 µl 1× PBS, of which 50 µl was transferred into a separate 96-well assay plate. For 3D hydrogel experiments, constructs were washed with 1× PBS and then homogenized in 200 µl 1× RIPA buffer, using a small pestle. Samples were then centrifuged to pellet the hydrogel material and cell debris. In duplicate, 50 µl of the supernatant was transferred into a separate 96-well assay plate. To measure ALP activity, 50 µl ALP substrate (Sigma) was then added to each well, and photometric absorbance at 405 nm was measured 10 times at 2 min intervals using a Wallac Victor^2^ plate reader (Ambler *et al.*, 1970). Relative ALP activity was calculated by taking the slope created when plotting absorbance at 405 nm vs time (min). The average slope of each duplicated sample was used for subsequent comparative analysis.

### 2.4. Calcium deposition assays

For 2D studies, 96-well plates used for ALP activity assays were subsequently used to measure the relative amount of calcium deposited on the plate surface. Briefly, the wells containing the remainder of the cell lysates were aspirated and washed with PBS. 100 µl 0.6 N HCl was then added to each well to dissolve deposited calcium. The plates were wrapped in parafilm and kept at 4 °C for at least 48 h. Samples were then diluted with 150 µl PBS. Calcium levels were measured by transferring 50 µl of the diluted sample to a separate assay plate and adding 100 µl of a photometric calcium-binding reagent (Pointe Scientific, Canton, MI USA) (Anderegg *et al.*, 1954; Baginski *et al.*, 1973). For 3D hydrogel experiments, 50 µl concentrated HCl was added to each sample after drawing off two 50 µl aliquots for ALP assays. The samples were vortexed briefly and then placed at 4 °C for at least 48 h. 2 µl samples were then taken from each tube and placed into a 96-well assay plate and diluted with 50 µl 1× PBS. 300 µl freshly prepared calcium binding reagent (Pointe Scientific) was then added to each well. The absorbance of each sample at 560 nm was measured using a Wallac Victor^2^ plate reader and compared to standards that were prepared with purified calcium phosphate (Sigma), using treatments and dilutions similar to those used to prepare experimental samples. The reported calcium values were relative to the mass/volume of calcium phosphate standards.

### 2.5. Collagen staining

Collagen staining was accomplished using reagents found in the Sircol Collagen Kit (BioColor, Carrickfergus, UK) (Liu *et al.*, 2007). Briefly, cell culture plates were washed with PBS and then incubated in red collagen dye reagent for 15 min. The wells were then washed thoroughly with PBS to remove unbound dye. Bound dye was then solublized with 50 µl alkali reagent, and sample absorbance was measured at 550 nm using a Wallac Victor^2^ platereader.

### 2.6. Quantitative RT–PCR

RNA was extracted from samples using Trizol (Sigma) according to the manufacturer's instructions. RNA samples were further purified by digestion with Turbo DNase (Ambion) to remove possible contaminating DNA that could interfere with analysis by qPCR. DNase activity was then removed from the samples by treatment with phenol, followed by salt–ethanol precipitation. The concentration and purity of the RNA obtained from this procedure was measured using a NanoDrop UV-Vis spectrometer. cDNA was prepared from total RNA harvested from tissue samples, using the iScript Synthesis kit (Bio-Rad, Hercules, CA, USA). A control reaction containing no reverse transcriptase was also performed to verify the absence of contaminating genomic DNA. Relative mRNA expression of *ALP, COL1A, CBFA1* and *OPN* was measured by quantitative real-time polymerase chain reaction (qPCR), using a BioRad iCycler. Standard curves were performed to ensure that primer efficiencies were within acceptable margins (90–110%). Expression of the housekeeping gene glyceraldehyde-3-phosphate dehydrogenase (*GAPDH*) was used as a loading control to normalize the relative levels of each mRNA in the experimental samples. Primer sequences used for qRT–PCR analysis are described in Table S1 (see Supporting information).

### 2.7. Statistical analysis

**Figure 1:**
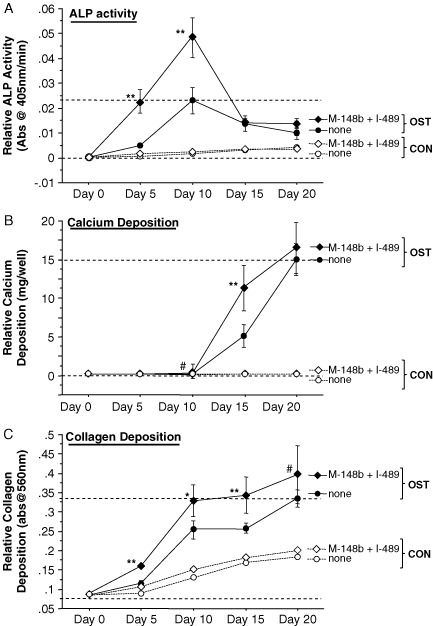
M-miR148b (M-148b) and I-miR489 (I-489) enhance the osteogenic activity of hMSCs. Transfected and non-transfected hMSCs were plated onto 2D surfaces and cultured for 20 days. Transfected hMSCs treated with OST medium showed an earlier and more robust onset of (A) alkaline phosphate (ALP) activity, (B) calcium deposition and (C) collagen accumulation. Dotted lines are included to indicate the minimal and maximal values of non-transfected controls treated with OST medium. Error bars represent 95% confidence intervals (CIs). When statistically significant, the synergistic interaction between miRNA transfection (M-miR148b + I-miR489) and OST medium treatment is marked by symbols representing the *p* value for this interaction: ^##^*p* < 0.05; *p < 0.01; **p < 0.001

**Figure 2:**
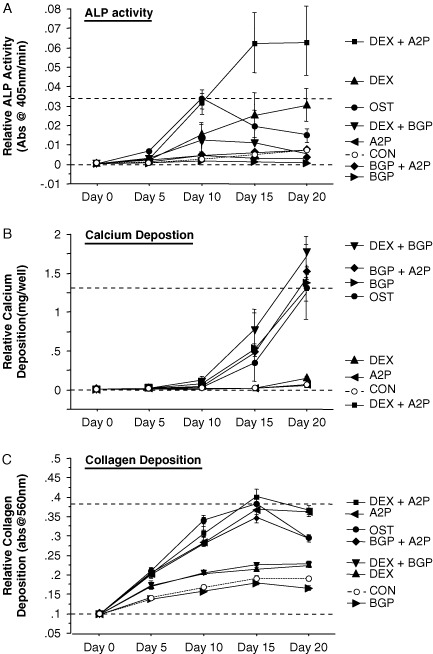
Individual components of OST medium were tested for their ability to induce (A) ALP activity, (B) calcium deposition and (C) collagen expression in 2D hMSC cultures. Treatment that included DEX had the greatest increases in ALP activity, BGP was required for calcium deposition and A2P had the greatest effect on collagen accumulation. The combination of these soluble factors (OST medium) showed unique patterns of these markers over the time course. Dotted lines are included to indicate the minimal and maximal values of non-transfected controls treated with OST medium. Error bars represent 95% CI

**Figure 3:**
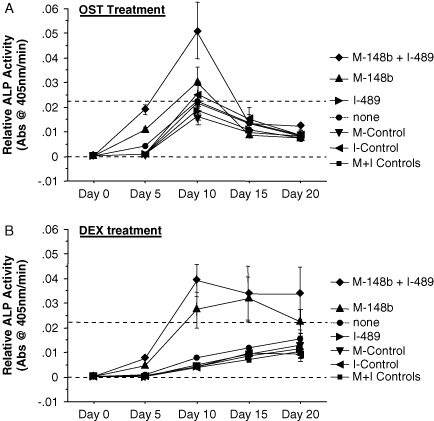
miRNA effect on ALP activity of hMSCs cultured on 2D surfaces and treated with either (A) OST medium or (B) DEX alone. Dotted lines are included to indicate the minimal and maximal values of non-transfected controls treated with OST medium. Error bars represent 95% CI

**Figure 4:**
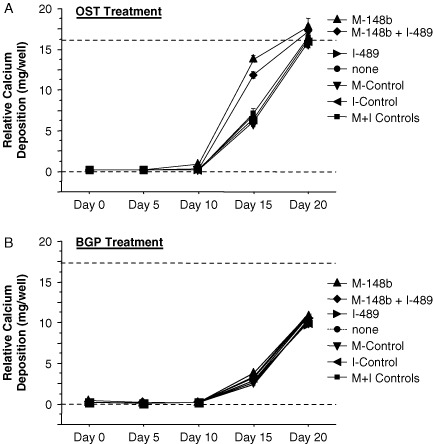
miRNA effect on calcium deposition of hMSCs cultured on 2D surfaces and treated with either (A) OST medium or (B) BGP alone. Dotted lines are included to indicate the minimal and maximal values of non-transfected controls treated with OST medium. Error bars represent 95% CI

**Figure 5:**
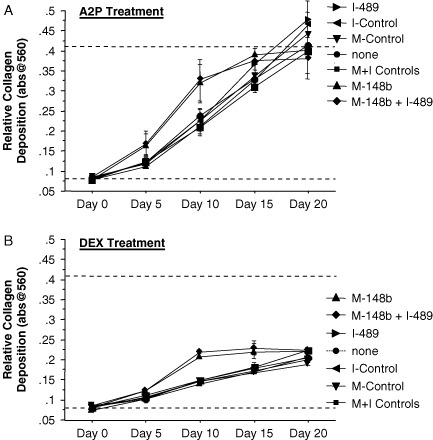
miRNA effect on collagen expression of hMSCs cultured on 2D surfaces and treated with either (A) A2P or (B) DEX alone. Dotted lines are included to indicate the minimal and maximal values of non-transfected controls treated with OST medium. Error bars represent 95% CI

**Figure 6:**
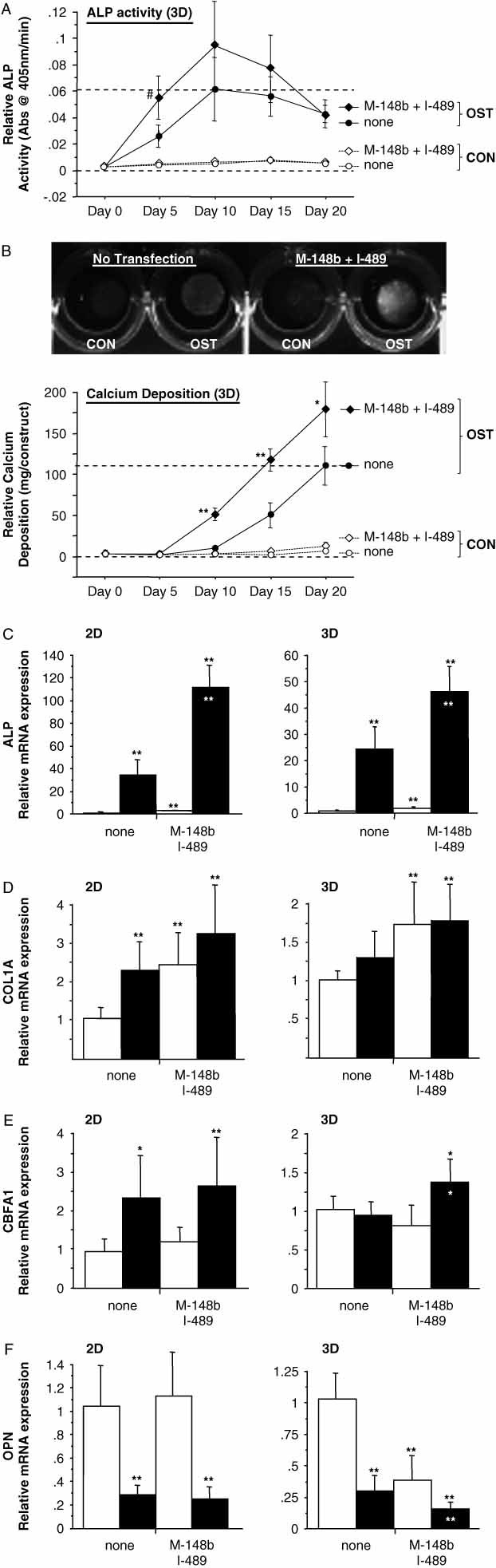
Transfection of hMSCs with M-miR148b (M-148b) and I-miR489 (I-489) enhances the osteogenic response to OST medium in 3D tissue constructs. miR-transfected hMSCs show early and robust responses in (A) ALP activity and (B) calcium deposition. Dotted lines are included to indicate the minimal and maximal values of non-transfected controls treated with OST medium. When statistically significant, the synergistic interaction between miRNA transfection (M-miR148b + I-miR489) and OST medium treatment is marked by symbols representing the *p* value for this interaction: ##*p* < 0.05; *p < 0.01; **p < 0.001. Gel images show representative constructs in 24-well plates at day 20 for each condition. (C) *ALP*, (D) *COL1A*, (E) *CBFA1* and (F) *OPN* mRNA levels from 3D constructs were quantified by qRT–PCR and are generally consistent with 2D expression. Expression levels were normalized to *GAPDH*. Media treatments are indicated by white bars (CON medium) and black bars (OST medium). The statistical significance between treatment groups and controls is marked with symbols. Also, when statistically significant, the synergistic interaction between miRNA transfection (M-miR148b + I-miR489) and OST medium treatment is marked by white symbols within the doubly-effected bars to identify the *p* value for this interaction: ^##^*p* < 0.05; *p < 0.01; **p < 0.001. All error bars represent 95% CI

The data presented in Figures 1–6 represent values pooled from independent experiments, with error bars used to designate 95% confidence intervals (CIs) (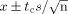). Statistical analysis (α = 0.05) was performed using a two-way ANOVA mixed-effects model, with media treatment and miRNA transfection as the main effects and interaction assumed. *Post hoc* multiple comparisons at each time point were made using a Fisher PLSD test. *p* values for critical comparisons are described in the text or figure legends.

## 3. Results

### 3.1. miRNA 148b mimic and 489 inhibitor accelerate osteogenesis in hMSC cultures

Although Schoolmeesters *et al.* (2009) showed that ALP activity was increased 6 days post-transfection, further characterization was needed to determine whether the miRNA mimics and inhibitors had lasting effects on cellular differentiation, specifically with respect to osteogenesis and the presentation of bone-related markers. To do this, hMSCs were transfected with a combination of both a mimic of miR-148b (M-miR148b) and an inhibitor of miR-489 (I-miR489) and allowed to differentiate in either CON medium or medium supplemented with soluble osteogenic factors (OST) in 2D culture plates. In order to confirm that the miRNA reagents effectively alter the expression levels of miR-148b and miR-489, sample plates were harvested after 48 h and analysed by qRT–PCR. Transfected hMSCs showed a > 100-fold increase in miR-148b levels, while miR-489 levels were undetectable (data not shown), suggesting that the transfection process effectively alters miRNA levels in these cells.

Over the course of 20 days, the hMSC cultures were harvested and analysed for standard bone-related markers. As shown in Figure 1A, miRNA transfection showed little effect on ALP activity in cultures treated with CON medium. In contrast, miRNA-transfected hMSCs treated with OST medium had increased ALP activity at days 5 and 10 compared with non-transfected cells in the same media conditions. These data suggest that M-miR148b and I-miR489 sensitized the hMSCs to the ostoegenic signals found in OST medium, resulting in quicker and/or more robust response to osteoinductive soluble factors.

This effect was also observed in the deposition of calcium, in that cultures containing miRNA-transfected hMSCs treated with OST medium demonstrated increased levels of calcium deposition over non-transfected cells at day 15. By day 15, increased calcium deposition was observed in OST-treated cultures, with miRNA-transfected cultures displaying > two-fold levels over non-transfected cultures. As can be seen in the plots, the calcium deposition in untransfected cultures appears to be catch up to transfected cultures, suggesting that they are reaching the same endpoint even though the initial rate of mineralization is different. No significant difference in calcium deposition was observed between transfected and non-transfected cells in CON medium.

As shown in Figure 1C, collagen expression was also affected by of hMSCs with M-miR148b and I-miR489. By Day 5, transfected cultures treated with OST medium had an increased accumulation of collagen compared with non-transfected controls. This effect remained through the course of the study, although the rate of collagen deposition appears to show similar trends to those of non-transfected cultures thereafter.

As shown in Figure 1, the combination of M-miR148b and I-miR489 transfection and OST medium treatment appeared to have a synergistic effect on multiple markers of osteogenic differentiation (ALP activity, calcium deposition, and collagen accumulation). This result was confirmed by statistical analysis using an appropriate ANOVA mixed-effects model, which showed that the combined effect of media treatment and miRNA transfection was significant (*p* < 0.05). *Post hoc* multiple comparisons allowed us to identify the time points where this synergistic effect occurred. These data are marked in each plot by symbols representing the statistical significance for this interaction.

### 3.2. Osteogenic media components differentially activate bone-related markers in hMSCs

Given that the transfection of hMSCs with M-miR148b and I-miR489 appeared to predispose the cells to osteogenic signals, resulting in a more robust induction of osteogenic markers in response to the additional soluble factors present in OST medium, we wanted to identify the individual contributions of each of these media supplements on standard bone-related markers. Traditionally, osteogenesis is induced in hMSC cultures by the addition of dexamethasone (DEX), β-glycerol phosphate (BGP) and ascorbic acid 2-phosphate (A2P) (Jaiswal *et al.*, 1997), so non-transfected hMSCs were treated with each of these additives alone or in combination. As shown in Figure 2A, hMSCs treated with media that included DEX had elevated ALP activity over controls in at least one of the time points measured, with the combination of DEX and A2P inducing the highest levels of ALP activity by the end of the time course. hMSCs treated with DEX and BGP (DEX + BGP, OST) had initial increases in ALP activity that reached a peak at day 10 before decreasing through day 20. hMSCs treated with either BGP or A2P, alone or in combination, showed no significant increase in ALP activity compared with untreated controls.

BGP treatment appeared to have the greatest impact on mineralization, in that all of the conditions that included BGP (BGP, BGP + A2P, DEX + BGP, OST) showed increased calcium levels over the control conditions (Figure 2B). In contrast, neither DEX nor A2P treatment appeared to have an effect on calcium deposition in hMSC cultures. These data suggest that BGP is both necessary and sufficient for increasing calcium deposition in hMSC cultures to levels that are associated with osteogenic differentiation. As shown in Figure 2C, A2P is clearly important to collagen deposition of hMSC cultures, as each treatment that contained A2P (A2P, DEX + A2P, BGP + A2P, and OST) showed elevated collagen staining over each of the conditions that did not include A2P. DEX and DEX + BGP also showed elevated collagen deposition over CON and BGP treatments, suggesting that DEX does increase collagen expression, although not to the extent of A2P (a derivative of vitamin C).

### 3.3. M-miR148b enhances DEX-mediated increase in ALP activity

hMSCs transfected with M-miR148b + I-miR489 and treated with OST medium showed increases in ALP activity at days 5 and 10 compared with non-transfected controls (Figure 1A). Given that a combination of miRNA mimics and inhibitors were used for this experiment, we were interested in knowing whether the miRNAs were contributing equally to this robust induction of ALP activity or whether one of the miRNA reagents, either M-miR148b or I-miR489, was having a dominant effect on the cells. To test this, hMSCs were transfected with either M-miR148b or I-miR489 and cultured in OST medium for 20 days. As shown in Figure 3A, hMSCs transfected with M-miR148b showed elevated ALP activity over controls at day 5, whereas hMSCs transfected with I-miR489 showed little difference in ALP activity over the course of the study. Although this would suggest that I-miR489 does not affect ALP activity in these osteogenic assays, the combination of M-miR148b + I-miR489 did result in a robust induction of ALP activity at both day 5 and day 10 when compared to cells transfected with M-miR148b alone. These data suggest that I-miR489 may work synergistically with M-miR148b, further enhancing its effect on ALP activity.

Because DEX proved to be the critical component of OST medium that induces ALP activity in hMSCs (Figure 2A), we hypothesized that M-miR148b transfection was effectively sensitizing the hMSCs to DEX-mediated signalling. As shown in Figure 3B, hMScs transfected with M-miR148b showed trends of ALP activity similar to those of cells transfected with the combination of M-miR148b and I-miR489, while hMSCs transfected with I-miR489 alone showed little difference in ALP activity when compared to non-transfected controls. These data are consistent with the results presented in Figure 3A and indicate that the transfection of hMSCs with M-miR148b acts to enhance the response to DEX, leading to elevated ALP activities at early time points.

### 3.4. M-miR148b increases the rate of calcium deposition in hMSCs treated with OST medium

The initial rate of calcium deposition in OST-treated hMSCs was increased in hMSCs transfected with M-miR148b + I-miR489 (Figure 1B), suggesting that these miRNAs accelerate bone-related mineralization of the extracellular environment. Since M-miR148b had the greatest effect on ALP activity (Figure 3), we hypothesized that the same would be true for calcium deposition. As shown in Figure 4A, hMSCs transfected with M-miR148b did accelerate the deposition of calcium that results from treatment of cultures with OST medium. Unlike its effect on ALP activity, in which the transfection of M-miR148b alone did not match cells transfected with both M-miR148b and I-miR489, M-miR148b transfection showed rates of calcium deposition similar to those of doubly-transfected cells. I-miR489 transfection appeared to have no effect on calcium deposition.

Because BGP proved to be the critical component of OST medium that induces calcium deposition in hMSCs (Figure 2B), we hypothesized that miRNA transfection was effectively sensitizing the hMSCs to BGP-mediated signalling. As shown in Figure 4B, this was not the case. Instead, neither M-miR148b nor I-miR489 caused an earlier onset of calcium deposition when compared to non-transfected and doubly-transfected hMSCs when treated with BGP alone. These data suggest that other components of OST medium, likely the presence of ALP-inducing DEX, are required for miRNA-mediated increases in calcium deposition.

### 3.5. M-miR148b increases the rate of collagen deposition in hMSCs treated with A2P or DEX

The amount of collagen deposition in OST-treated hMSCs was increased in hMSCs transfected with M-miR148b + I-miR489 (Figure 1C), suggesting that these miRNAs accelerated collagen expression of osteogenic hMSCs. Because the treatment of hMSC with A2P or DEX proved to be the critical components of OST medium that induced collagen expression (Figure 2C), we examined the roles of each miRNA individually in A2P and DEX to identify which miRNA reagents were contributing to this effect. As shown in Figure 5A, hMSCs transfected with M-miR148b showed a similar pattern of collagen deposition in as the doubly-transfected cells when treated with A2P alone. A similar effecet was observed in DEX-treated cultures (Figure 5B). These data indicate that M-miR148b, not I-miR489, plays a critical role in increasing collagen deposition in response to osteogenic media.

### 3.6. miRNA transfection increases osteogenic markers in 3D tissue scaffolds

The ultimate goal of this study was to evaluate the potential of miRNA-based strategies for enhancing osteogenic differentiation of hMSCs for tissue-engineering purposes. 2D studies were employed to quickly gain an understanding of the effects of M-miR148b and I-miR489 on osteogenic markers in hMSC cultures (Figures 1–5) and direct the development of biomaterials for miRNA-based strategies. While these studies clearly demonstrate that these miRNA reagents act to sensitize hMSCs to soluble osteogenic signals, it remained to be seen whether these effects would also be seen when the cells were placed in 3D scaffolds that more closely mimic a natural tissue environment. Therefore, hMSCs were transfected and then encapsulated in PEG-based tissue scaffolds. ALP activity and calcium deposition in these constructs was monitored for 20 days. As shown in Figures 6A, B, miRNA transfection clearly enhanced ALP activity and calcium deposition in 3D tissue constructs. These results indicate that our results from 2D tissue culture (Figures 1–5) are predictive of 3D culture.

To further establish the validity of 2D tissue culture results and confirm that miRNA transfection enhances the onset of osteogenesis in these 3D constructs, qRT–PCR was employed to measure the expression levels of key osteogenic markers in our hydrogels. The day 5 time point was chosen for RNA harvest because it provided an opportunity to determine whether miRNA transfection had caused an earlier expression of bone-related genes. qRT–PCR was performed on hMSCs cultured in 2D culture plates as a point of reference to confirm that the 3D constructs were comparable to previous results.

A significant difference was observed in ALP expression on day 5 between non-transfected and miRNA-transfected hMSCs treated with CON medium for both 2D and 3D cultures (Figure 6C). These data were consistent with ALP activity assays presented in Figure 1A and indicate that *ALP* mRNA expression is a highly predictive measure of enzyme activity in this system. Like the 2D ALP activity assays presented in Figure 1A, the increase in ALP activity was relatively small when compared to the increase observed in OST-treated cells, an effect that is enhanced in miRNA-transfected cells.

hMSCs transfected with M-miR148b and I-miR489 showed significant increases in collagen 1A (COL1A) expression, independent of soluble osteogenic factors (OST medium; Figure 6D). Transfected cells cultured in CON medium showed increases in COL1A expression at day 5, suggesting that these miRNA reagents alone have some osteoinductive potential. COL1A expression was also elevated in miRNA-transfected hMSCs cultured in OST medium, although significant differences were not observed between transfected and non-transfected cells under these conditions.

A difference in CBFA1 expression was observed between 2D and 3D culture formats (Figure 6E). In 2D cultures, OST medium increased *CBFA1* mRNA expression, although no significant difference was observed between transfected and non-transfected cells. In 3D, however, only constructs containing miRNA-transfected hMSCs showed elevated levels of *CBFA1* mRNA.

Measured mRNA levels of osteopontin (OPN), a well-established marker of mature bone, were unexpected, with significant decreases in OPN being observed in cells treated with OST medium (Figure 6F). In 2D culture, nearly identical expression levels were observed between transfected and non-transfected hMSCs, suggesting that these miRNA reagents had no effect on OPN expression. In contrast, significant differences were observed between transfected and non-transfected cells in 3D hydrogels. Transfected cells had lower OPN expression levels in CON medium, an effect that was also observed in OST medium. Although in an unexpected direction, miRNA transfection had the effect of enhancing the effects of osteogenesis induced by OST medium.

## 4. Discussion

Finding ways to improve the rate of tissue regeneration is crucial to the development of successful therapeutic strategies aimed at repairing bone mass deficiencies. While patent-derived stem cell therapies provide a promising strategy for increasing tissue regeneration without the risk of immune rejection, the efficient induction of osteogenic differentiation of delivered stem cells remains an aspect crucial to the success of stem cell-based therapies. In the work presented here, we demonstrate that the use of a miR-148b mimic and an miR-489 inhibitor has the effect of sensitizing hMSCs to soluble osteogenic factors, resulting in the rapid and robust induction of bone-related markers.

miRNA transfection had the effect of both increasing the amplitude of the osteogenesic response to soluble factors as well as accelerating its progression. In the case of ALP activity, miRNA transfection increases the amplitude of the response, effectively increasing ALP levels at an earlier timepoint than non-transfected cells. As shown in Figure 1, ALP activity is significantly reduced after day 10 in both transfected *and* non-transfected hMSC cultures. ALP activity is likely downregulated as the cultures differentiate and mature. Of note, ALP activity is only downregulated in cultures that also contain BGP and begin to show signs of mineralization. This suggests that a feedback loop is initiated once mineralization begins and/or reaches critical levels. Although calcium deposition is only minimally higher in miRNA-transfected cells in 2D cultures on day 20, two important points in the data we present should be considered: (a) calcium deposition in 2D culture is higher in transfected cultures on day 15, suggesting that mineralization in these cultures has either initiated earlier or progressed more rapidly than non-transfeceted cultures; and (b) calcium deposition is significantly higher in 3D hydrogels containing transfected cells. In the case of mineralization, it appears that miRNA transfection acts to reduce the time it takes to arrive at the same point as non-transfected cells, suggesting that the cultures are maturing more quickly but arriving at the same endpoint. The fact that the 3D constructs continue to mineralize suggests that they have not reached a maximal level within 20 days, which stands to reason given the increased volume available for calcium deposition.

Interestingly, OPN levels are down-regulated in MSCs within the first 5 days of osteogenic differentiation (Figure 6F). At first glance, this would suggest that miRNA transfection was acting in opposition to osteogenesis, because OPN is considered a standard marker of osteoblasts. It must be kept in mind, however, that we purposely chose to examine an early time point in order to evaluate the ability of these miRNAs to speed up osteogenesis. At day 5, even non-transfected cells treated with OST medium show a significant decrease in *OPN* mRNA levels. As with the other gene markers, transfection with miRNAs acted to enhance the effect of OST medium. Why OPN is downregulated during the early stages of ostegenesis in hMSCs is unclear, but the ability of the miRNA mimics and inhibitors to enhance this effect is obvious. mRNA levels of *OCN* were also downregulated at day 5, but transfection with miRNAs appeared to have no effect on OCN expression (data not shown), suggesting that OPN and OCN, both markers of mature bone tissue, are differentially regulated. By day 20, no significant differences in mRNA levels of each of the genetic markers *(ALP, COL1A, CBFA1, OPN*, and *OCN*) were observed between transfected and non-transfected hMSCs under the same media conditions (data not shown). This is to be expected, since the endpoint, the completion of osteogenesis, should be the same. If differences had been observed between transfected and non-transfected cultures, questions would remain about the long-term effects of miRNA transfection.

When compared to other nucleic acid-based strategies for controlling behaviour, the use of miRNAs has many advantages. miRNAs naturally function to control differentiation in cells; therefore, targeting their activity is a relatively direct way to control cell fate. Moreover, miRNAs are short RNA molecules that are relatively easy produce and introduce into mammalian cells, which is not the case for plasmid or viral strategies. Plasmid transfection involves the introduction of large DNA molecules, which often suffer from low transfection efficiencies, especially in primary cells such as hMSCs. Viral infection results in high gene transmission efficiency but requires time-consuming vector preparation and carries with it the stigma of viral-mediated infection, presenting a significant obstacle for clinical applications.

Furthermore, miRNAs function in the cytoplasm and do not present a risk of adverse genetic effects. Although previous studies have shown that MSC differentiation can be enhanced by the over-expression of either BMP-2 or CBFA1, over-expression techniques involve the incorporation of complete transcriptional units that will have the potential to result in unknown genetic transmission effects. Although the likelihood of this happening is extremely small, genetic engineering of this sort is generally approached with hesitation and would probably lead to prolonged delays in testing before, if ever, becoming available for clinical use.

The vast potential of targeting miRNA activity in cells is likely to result in an explosion of miRNA-based approaches in the field. As is becoming increasingly clear, miRNAs are global regulators of cell function and are intimately involved in controlling cell phenotypes. For this reason, their use for directing tissue regeneration could be applied to all tissue types, not just bone. In fact, a recent study by Rhim *et al.* (2010) shows that inhibiting the activity of miR-133 in C2C12 myoblasts acts to enhance the muscle properties of bioartificial tissue constructs, indicating that miRNA-based approaches could be applied to a wide range of tissue-regeneration applications, including muscle. Research has indicated that every cell type in the body has its own unique miRNA expression profile, suggesting that it would be possible to identify specific miRNA mimics and inhibitors that could be used to direct cellular differentiation toward any number of tissues. Similarly, the manipulation of miRNA activity could be used to direct cells away from unwanted disease phenotypes.

The work presented here focuses on a tissue-engineering approach that involves the transfection of hMSCs with specific miRNA mimics and inhibitors in order to direct their differentiation. For clinical applications, transfected cells could either be delivered directly to the patient or returned to tissue culture incubators in 2D plates or 3D tissue scaffolds for maturation before implantation into the patient. While these would certainly be feasible options, the use of miRNAs for tissue-engineering purposes could be accomplished by a variety of means. For instance, miRNA reagents (mimics, inhibitors or other activators/repressors of miRNA activity and function) could be delivered directly to areas of interest within the body in order to affect the activity of local cells. In the case of biomaterials scaffold strategies, such as the hydrogels presented here, miRNA-targeting reagents could be incorporated into the scaffold as a way to both localize these signals and/or control their release to the surrounding tissue or invading cells.

## Supporting information on the internet

**Table S1.** Primer sequences used for qRT–PCR
