# Supplementary material for: Manipulation of miRNA activity accelerates osteogenic differentiation of hMSCs in engineered 3D scaffolds
Source: J Tissue Eng Regen Med. 2011 Jun 27;6(4):314–24. doi: 10.1002/term.435 (PMC3184319; doi:10.1002/term.435)
Supplement: Supplementary file 1 [file term0006-0314-SD1.ppt]

## Slide 1
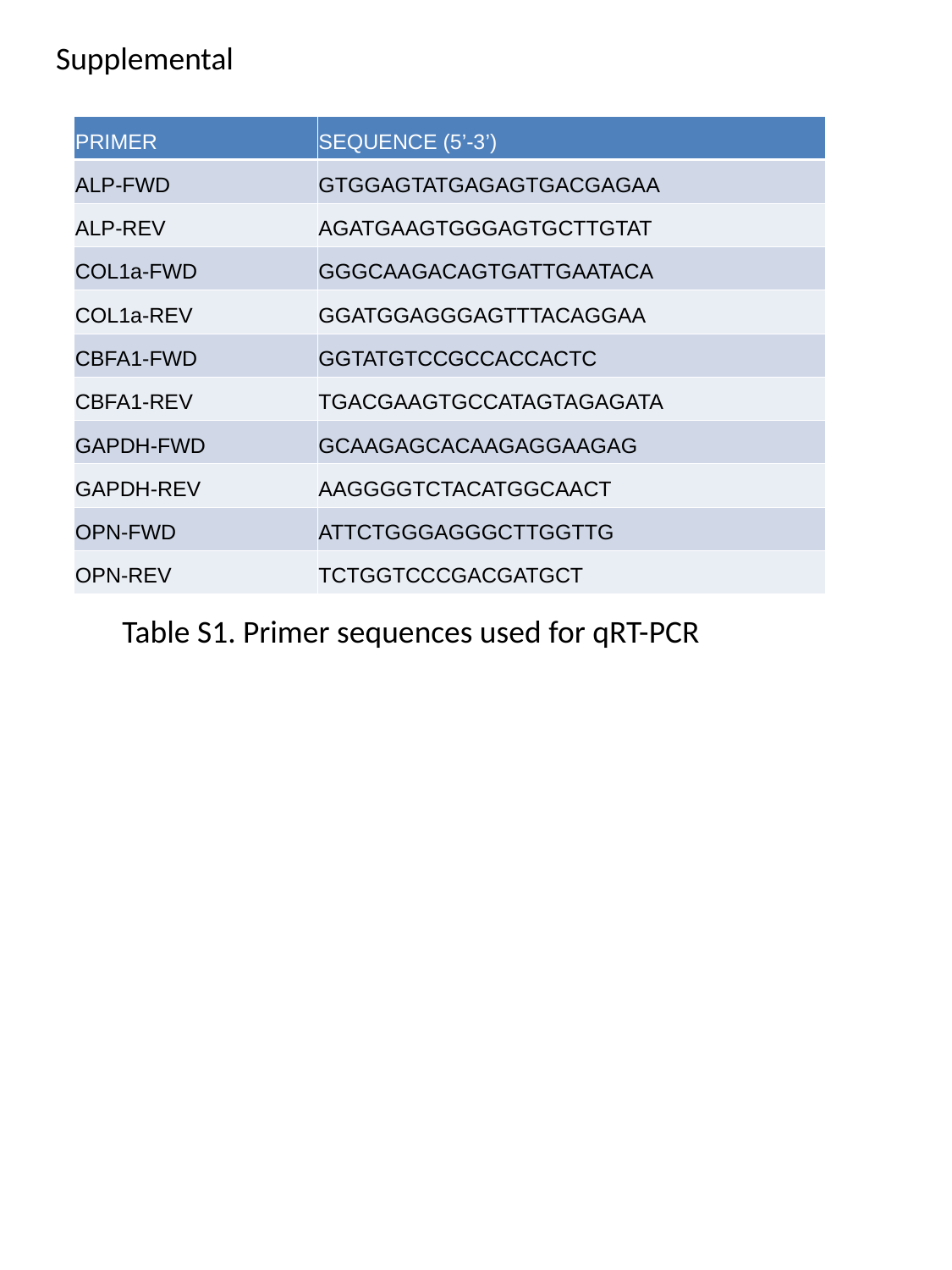

Supplemental
| PRIMER | SEQUENCE (5’-3’) |
| --- | --- |
| ALP-FWD | GTGGAGTATGAGAGTGACGAGAA |
| ALP-REV | AGATGAAGTGGGAGTGCTTGTAT |
| COL1a-FWD | GGGCAAGACAGTGATTGAATACA |
| COL1a-REV | GGATGGAGGGAGTTTACAGGAA |
| CBFA1-FWD | GGTATGTCCGCCACCACTC |
| CBFA1-REV | TGACGAAGTGCCATAGTAGAGATA |
| GAPDH-FWD | GCAAGAGCACAAGAGGAAGAG |
| GAPDH-REV | AAGGGGTCTACATGGCAACT |
| OPN-FWD | ATTCTGGGAGGGCTTGGTTG |
| OPN-REV | TCTGGTCCCGACGATGCT |
Table S1. Primer sequences used for qRT-PCR
